# Supplementary material for: The Dipeptidyl Peptidase-4 Inhibitor Linagliptin Ameliorates Endothelial Inflammation and Microvascular Thrombosis in a Sepsis Mouse Model
Source: Int J Mol Sci. 2022 Mar 12;23(6):3065. doi: 10.3390/ijms23063065 (PMC8949150; doi:10.3390/ijms23063065)
Supplement: Supplementary file 1 [file ijms-23-03065-s001.zip › ijms-1597349-supplementary.pdf]

Supplementary Data

Cell Viability Assay

Cell viability were analyzed by Cell Counting Kit-8 (Dojindo Molecular Technologies). HUVECs were seeded in 48-well. The medium were changed after 2 days and treated with linagliptin for 48 h. Adding 25 μL of the CCK-8 solution to the medium and then Incubating the plate for 2 h in the incubator (5% CO_2, 37 ℃). Transfer 100 μL medium containing CCK8 solution to the 96-well plate and measure the absorbance at 450 nm by ELASA plate reader (TECON).

Table S1. The list of antibodies used for western blot

Antibody	2° antibody	Dilution	Source
p- eNOS (ser-1177)	Rabbit	1:1000	Cell signaling #9571
eNOS	Rabbit	1:1000	Santa Cruz sc-8311
p-Akt (ser-473)	Rabbit	1:1000	Cell signaling #9272
Akt	Rabbit	1:1000	Cell signaling #9271
β-actin	Mouse	1:5000	Sigma Aldrich A5316
Lamin B	Goat	1:500	Santa Cruz sc-6217

Table S2. Primer sequences for qRT-PCR. (Human)

Gene	Primer Sequence (5′→3′)
ICAM1	Forward: ATGCCCAGACATCTGTGTCC<br>Reverse: GGGGTCTCTATGCCCAACAA
VCAM1	Forward: GGGAAGATGGTCGTGATCCTT<br>Reverse: TCTGGGGTGGTCTCGATTTTA
IL6	Forward: ACTCACCTCTTCAGAACGAATTG<br>Reverse: CCATCTTTGGAAGGTTCAGGTTG
IL1B	Forward: ATGATGGCTTATTACAGTGGCAA<br>Reverse: GTCGGAGATTCGTAGCTGGA
RELA (p65)	Forward: ATGTGGAGATCATTGAGCAGC<br>Reverse: CCTGGTCCTGTGTAGCCATT

Table S3. Primer sequences for qRT-PCR. (Mice)

Gene	Primer Sequence (5′→3′)
ICAM1	Forward: GTGATGCTCAGGTATCCATCCA<br>Reverse: CACAGTTCTCAAAGCACAGCG
IL6	Forward: CCAGAGATACAAAGAAATGATGG<br>Reverse: ACTCCAGAAGACCAGAGGAAAT
IL1B	Forward: GAAATGCCACCTTTTGACAGTG<br>Reverse: TGGATGCTCTCATCAGGACAG
RELA (p65)	Forward: AGGCTTCTGGGCCTTATGTG<br>Reverse: TGCTTCTCTCGCCAGGAATAC
iNOS	Forward: GTTCTCAGCCCAACAATACAAGA<br>Reverse: GTGGACGGGTCGATGTCAC
TNF	Forward: CCCTCACACTCAGATCATCTTCT<br>Reverse: GCTACGACGTGGGCTACAG

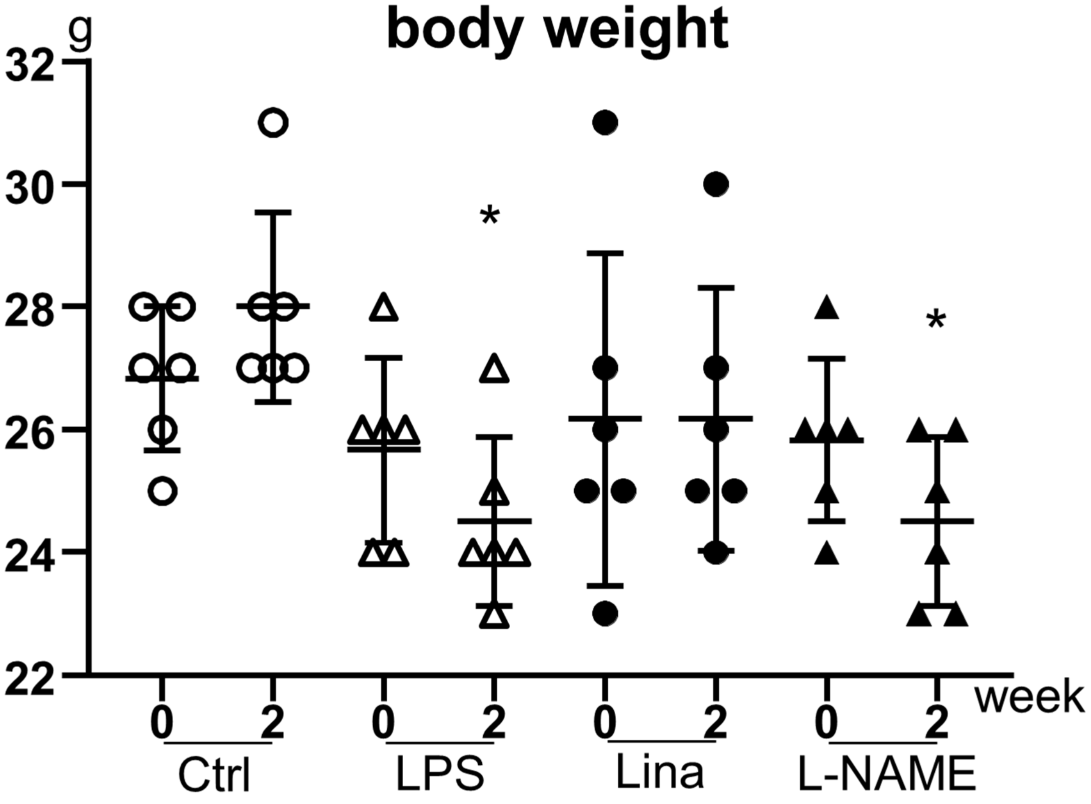

Figure S1. Mice body weight changes after LPS injection. For Linagliptin-treated mice, Linagliptin was fed on day 12th and 13th. For L-NAME-preteated mice, 0.25g L-NAME was added into drinking water 250ml (concentration: 3.7mM) through the whole experiment and Linagliptin was fed on day 12th and 13th. For LPS, Linagliptin-treated group, and L-NAME preteated mice, the LPS was injected into the mice on day 13th. Mice was sacrificed on day 14th. The body weight was measured at the beginning and on day 14th. * $p < 0.05$ compared with control group body weight of corresponding time. (Ctrl: control group; LPS: LPS group; Lina: Linagliptin-treated group; L-NAME: L-NAME pretreated group; 0: at the beginning of experiment; 2: 2weeks after the experiment).
